# The relationship between stigma and quality of life in hospitalized middle-aged and elderly patients with chronic diseases: the mediating role of depression and the moderating role of psychological resilience

**DOI:** 10.3389/fpsyt.2024.1346881

**Published:** 2024-05-21

**Authors:** Qiqi Ji, Lin Zhang, Jiashuang Xu, Pengjuan Ji, Miaojing Song, Yian Chen, Leilei Guo

**Affiliations:** ^1^ School of Nursing, Jinzhou Medical University, Jinzhou, Liaoning, China; ^2^ Department of Internal Medicine Nursing, School of Nursing, Wannan Medical College, Wuhu, An Hui, China

**Keywords:** middle-aged and elderly, stigma, depression, quality of life, chronic disease

## Abstract

**Objective:**

Patients with chronic diseases may have some psychological problems due to their own or surrounding environmental factors, which can adversely affect the patient’s illness and life. Given that the number of chronically ill patients in China is currently increasing every year, more research is needed to determine the best ways to manage changes in psychological status and psychological stress responses in chronically ill patients. The researchers constructed a mediated moderation model to explore the impact of stigma on the quality of life of chronically ill patients, as well as the mediating role of depression and the moderating role of psychological resilience.

**Methods:**

A stratified sampling method was used to select 363 middle-aged and old-aged patients with chronic diseases aged 45 years and older from the Affiliated Hospital of Zhejiang University for the study. Data were collected from patients with chronic diseases such as cardiac, respiratory, renal, and other chronic diseases using the Cumulative Illness Rating Scale for Geriatrics (CIRS-G), the Stigma Scale for Patients with Chronic Diseases (SSCI), the Patient Health Questionaire-9 (PHQ-9), the Quality of Life Inventory (SF-12), and the Conner-Davidson Resilience Scale (CD-RISC) were collected from patients with cardiac, respiratory, renal, and other chronic diseases. A descriptive analysis was used to describe the sample. Linear regression was used to evaluate the relationship between the variables. Mediation and moderation analyses were used to explore the mediating role of depression and the moderating role of psychological resilience.

**Results:**

There was a moderate negative correlation between stigma and quality of life (*r* = -0.378, *P* < 0.01). There was a moderate negative correlation between depression and quality of life (*r* = -0.497, *P* < 0.01). There was a moderately positive correlation between psychological resilience and quality of life (*r* = 0.382, *P* < 0.01). There was a moderate negative correlation between psychological resilience and depression (*r* = -0.348, *P* < 0.01). There was a weak negative correlation between psychological resilience and stigma (*r* = -0.166, *P* < 0.01). There was a strong positive correlation between stigma and depression (*r* = 0.607, *P* < 0.01) The mediation study showed that stigma was a significant predictor of quality of life and that stigma and quality of life were mediated to some extent by depression, with the mediating effect accounting for 67.55% of the total effect. The direct path from stigma to depression is moderated by psychological resilience (*β* = -0.0018, *P* < 0.01).

**Conclusions:**

Depression mediates the relationship between stigma and quality of life, while psychological elasticity plays a moderating role between stigma and depression, and when the level of psychological elasticity increases, the more significant the role of stigma on depression. As a physiologically and psychologically vulnerable group, patients with chronic diseases’ overall quality of life and mental health should be taken more seriously, and clinical workers should pay timely attention to the psychological and mental conditions of patients with chronic diseases and provide timely and appropriate interventions and therapeutic measures. The relevant results of this study also provide a new perspective for clinical work on psychological intervention for patients with chronic diseases.

## Background

Chronic non-communicable diseases (often referred to as chronic diseases) are an abbreviation for a group of diseases that are not contagious but have an insidious onset, a long treatment period, and incurable (1). In the modern world, chronic diseases are a significant hazard to human health, accounting for almost 36 million deaths annually worldwide (2). The incidence of chronic diseases has progressively tended to increase in recent years as living standards have increased, with middle-aged and older individuals now making up the majority of those with chronic diseases (3). Patients with chronic diseases who are treated at home may decide to go to the hospital if their conditions worsen or if they are having an acute attack because of the wide range of causes of these illnesses and the difficulty of treating them (4, 5).

Quality of life is not only a way of evaluating an individual’s awareness of and satisfaction with his or her living conditions and status under different cultural backgrounds and customs (6), but also a comprehensive indicator reflecting an individual’s somatic function, mental health status, and social adaptability. The quality of life of patients with chronic diseases is affected by a variety of factors, among which the psychological factors of patients are very important to the quality of life (7). During the hospitalization of middle-aged and old-aged patients with chronic diseases, due to the special nature of the treatment environment, frequent medication, and heavy economic burden, all these will increase the burden of the patient’s self-feeling, resulting in the middle-aged and old-aged patients with chronic diseases hospitalized in the hospital triggering a stress response after enduring great physical and mental pressure and the emergence of negative psychology such as a sense of shame of the disease (7, 8). The stigma was first coined in 1963, and it refers to a sense of shame, meaning the transformation of an intact normal person into a person contaminated by something else, hence the term stigmatization (9, 10). As a negative psychological phenomenon, stigma can adversely affect treatment adherence and disease self-management in patients with chronic diseases (10). It leads to a loss of belief in disease treatment, and if these patients are not intervened within time, it will increase the difficulty of treating chronic diseases and seriously affect their quality of life (11, 12). It has been shown that the sense of shame, as a kind of negative psychology, has a negative correlation with the quality of life. Therefore, studying the contents related to the sense of shame and quality of life in middle-aged and elderly patients with chronic diseases can help to improve their quality of life (13).

Depression as a mental disorder is an important indicator to evaluate the mental health of the individual. According to the Fifth Edition of the Diagnostic and Statistical Manual of Mental Disorders (DSM-5) and the International Statistical Classification of Diseases and Related Health Problems (ICD-11) judgment criteria, individuals with depression, such as mental disorder symptoms, are due to a variety of reasons, including persistent low mood and reduced interest in life as the main clinical manifestations (14–16). Previous studies have shown (13, 17) that chronic disease patients suffering from depression have a lower level of quality of life. Depressive symptoms can exacerbate the chronic disease patients themselves and make treatment more difficult, thus leading to a lower quality of life (18). At the same time, people with chronic illnesses who suffer from depression lose interest in life and work, have fewer frequent social interactions, and have less interaction with their families, which ultimately harms their quality of life (19, 20).

The capacity for psychological resilience is the capacity for an individual to exhibit positive psychology in the face of adversity or trauma (21, 22). Psychological resilience can mitigate the psychological trauma brought on by negative emotions and chronic diseases, increase an individual’s adaptive capacity, improve their quality of life and subjective well-being, and lessen negative emotions, according to certain studies (23, 24). Additionally, when dealing with the obstacles and challenges of their condition, it can assist people with chronic diseases in being more motivated to receive treatment (25). A study by Chinese scholars reported (26)that there was a significant correlation between psychological resilience and illness stigma. Patients with a high level of psychological resilience can help maintain a stable psychological and physiological state in unfavorable environments, promote more active participation in social activities, buffer the psychological sense of shame that occurs in patients as a result of the disease, and be more accepting of changes in somatic functioning as a result of the disease, thus reducing the emergence of other negative emotions.

According to the biopsychosocial point of view, there is a complex interaction between biological, psychological, and social factors of the sick individual, and we should pay attention not only to the disease state of the sick individual but also to the condition of the sick individual itself (27, 28). Recognizing that the influences on an individual’s quality of life are numerous and interconnected, the biopsychosocial perspective is to manage effective quality of life as a primary therapeutic goal. It can have a variety of effects by concentrating on teaching people how to effectively raise their quality of life; these changes could include elevated moods, higher activity levels, better relationships, etc. Thus, it is advised that future healthcare providers begin from a biopsychosocial perspective when focusing on enhancing the quality of life of middle-aged and elderly patients with chronic conditions to better draw up a treatment plan for the patients.

**Figure 1:**
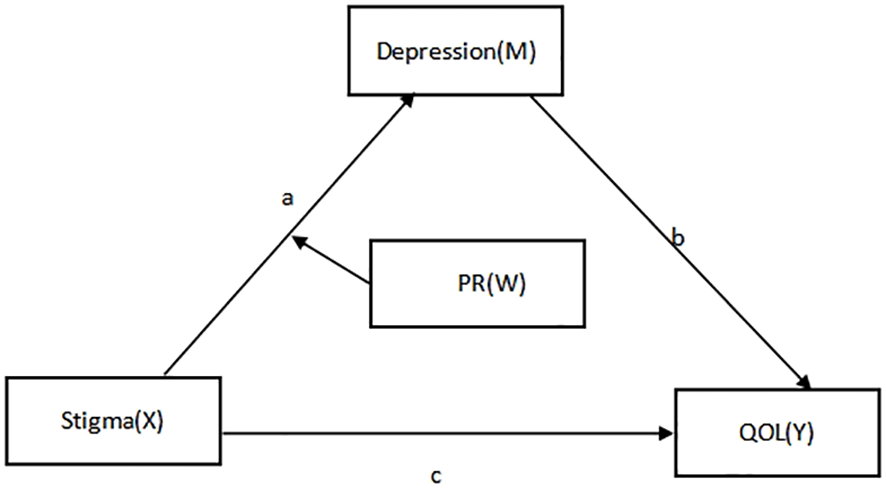
Hypothetical diagram of the model of the relationship between stigma, psychological resilience, depression, and quality of life.

As such, we put out a theoretical model to investigate the interaction among several factors to further understand the relationship between psychological resilience, stigma, depression, and quality of life. The following are the study’s hypotheses: H1: The quality of life is negatively correlated with stigma. H2: Depression mediates the relationship between stigma and quality of life. H3: Psychological resilience plays a moderating role in the relationship between stigma and depression (**Figure 1**).

## Materials and methods

### Participants and data collection

The study included participants who met four requirements: (1) could not have been younger than 45 years old; (2) had no documented history or diagnosis of neurological or psychiatric diseases (1); (3) could communicate well and had no comprehension difficulties; and (4) volunteered to participate in the study. Exclusion criteria: (i) patients with serious psychiatric disorders who are unable to communicate and express themselves normally; (ii) patients who did not cooperate with this investigation (3). The researcher employed the cross-sectional questionnaire method, followed informed consent guidelines, distributed and promptly returned the questionnaires, and used a uniform questionnaire guideline after receiving approval from the hospital’s ethical review committee and the pertinent departments.

### Sampling and sample size

Firstly, a randomly selected tertiary hospital from the Medical College of Zhejiang University was selected. Second, the departments of traditional Chinese medicine, endocrinology, and dermatology were randomly selected from the selected hospitals. Third, survey points were set up within the department to randomly select survey respondents. Ultimately, 378 research participants who satisfied the inclusion requirements had in-person conversations. According to Kendall’s criteria, the sample size is 5–10 times the number of items (29), and to increase the sample attrition rate by 10%, we need a total of 350 samples. Finally, 378 questionnaires were distributed, and 363 valid questionnaires were recovered, with an effective recovery rate of 96.03%.

### General information questionnaire

Following a thorough examination of a substantial body of literature, the researcher’s design was determined by the study’s objectives, which mostly covered age, gender, employment, place of residence, degree of personal health, and the existence of chronic pain.

### Cumulative illness rating scale-geriatrics

William L. Leidy et al. developed this scale in 1980 to evaluate an individual’s organ system-specific disease prevalence and severity (30). Thirteen items make up the four-point scale (0 being not present, 1 being mild, 2 being severe, 3 being more severe, and 4 being extremely severe). The degree of co-morbidity increases as the score rises. To better understand the health state and illness-related situations of middle-aged and older individuals, the scale was solely utilized in this study to assess the disease status of particular organ systems in these individuals.

### Stigma scale for chronic illness

Rao and other academics assembled the scale, which our scholars Deng Cuiyu et al. translated into Mandarin in 2017 (31). The 24-item measure gauges the level of internal and external stigma experienced by individuals with chronic diseases and includes both created and perceived stigma aspects. Higher scores on the scale indicate a higher level of stigma experienced by the patient. The scale was rated on a 5-point Likert scale from 1 (never) to 5 (always). The total score can range from 24–120. The Cronbach’s alpha coefficient for the scale in this study was 0.806, and the structural validity was 0.956.

### Patient health questionaire-9

The measure was created in the 1980s by Columbia University in the United States and is capable of both diagnosing depression in individuals and determining the severity of the illness (32, 33). This scale is one-dimensional and has nine entries. With a total score ranging from 0 to 27, higher scores indicate more severe depressive symptoms. The scale is scored on a 4-point Likert scale from 0 (not at all) to 3 (nearly every day). There were four grades used to describe depression levels: mild (6–9 points), moderate (10–14 points), severe (15–21 points), and very severe (22–27 points). The Cronbach’s alpha coefficient for this scale in this study was 0.852, and the structural validity was 0.880.

### Quality of life scale (SF-12 health survey)

The scale was compiled by the Boston Health Research Institute in the United States after being simplified based on the SF-36 scale and was translated and standardized by Zhejiang University in 1991 for promotion in China (34). The measure has twelve items and eight dimensions: mental health (MH), role emotional (RE), bodily pain (BP), general health (GH), vitality (VT), social functioning (SF), role physical (RP), and physical functioning (PF) (35, 36). The Mental Component Summary (MCS) is the total score of the last four dimensions on the scale, and the Physical Component Summary (PCS) is the total score of the first four dimensions on the scale. The patient’s quality of life is measured on a scale of 0 to 100, where higher scores correspond to better quality. The Cronbach’s alpha coefficient for this scale in this study was 0.795, and the structural validity was 0.832.

### Conner-Davidson resilience scale

The American academician’s Conner and Davidson created the measure, which has 25 items across five domains (mental affect, control, positive acceptance of change, acceptance of negative sentiments, and personal competency) (37). Higher scores indicate higher levels of psychological resilience. The measure is based on a 5-point Likert scale, with values ranging from “very inconsistent” to “very consistent” on a scale of 0–4. The total score ranges from 0 to 100. In this survey, the scale had a Cronbach’s alpha coefficient of 0.859 and a structural validity of 0.861.

### Statistical analyses

To analyze and process the data, the study employed SPSS 25.0 and Process 3.3 plug-ins (38, 39). ANOVA and t-tests were used to compare demographic differences in quality of life; Pearson correlation analysis was used to investigate the relationship between psychological resilience, depression, morbidity, shame, and sleep quality; measurements were expressed as mean ± SD and counts as the number of cases and percentages. A mediation model was developed using stigma as the independent variable (X), depression as the mediate (M), Psychological resilience as the moderate variable (W), quality of life as the dependent variable (Y), and general demographic information as the control variable to explore the direct pathway effect of stigma on quality of life (Stigma→Quality of life), and we utilized the Hayes’ (2013) process macros (Model 4) The mediating role of depression was evaluated (Stigma→Depression→Quality of life). Finally, we analyzed the moderation-mediation model using Hayes’ process macros (Model 7). A simple slope test (Mean ± SD) was used to illustrate the relationship between stigma and depression in middle-aged and elderly chronic disease in patients with high and low psychological resilience. The bootstrap method produces 95% bias-corrected CIs for these effects from 5000 re-samples of the data. CIs that do not contain zero indicate a significant effect (3).

## Results

### Descriptive statistics

Data were analyzed on the general demographic characteristics of the study population and the quality of life of the different characteristics. Of the 363 middle-aged and elderly chronic disease patients, 226 (62.70%) were male and 137 (37.30%) were female. The age range of the middle-aged and elderly chronic disease inpatients was 45–96 years, with a mean age of 63.86 ± 10.05 years. The quality of life level of middle-aged and elderly chronic disease inpatients varied across occupations and education levels, and these differences indicated that chronic disease patients with stable jobs and high education levels had higher quality of life levels. The relevant results are shown in **Table 1**. In addition, the chronic diseases that were more prevalent in this study were blood vessels, liver, endocrine/Metabolic, and breast cancer. The distribution of chronic diseases in different organ systems is shown in **Table 2**.

**Table 1:** One-way ANOVA in middle-aged and older patients hospitalized with chronic diseases.

Variable	Group	N (%)	Score	*F/t*	*P*
Sex	Men	226 (62.3%)	75.63 ± 9.59	0.004	0.947
	Women	137 (37.7%)	75.70 ± 10.94		
Careers	Workers	57 (15.7%)	79.16 ± 8.58		
	Office-bearer	10 (2.8%)	79.54 ± 7.53		
	Farmers	100 (27.5%)	73.85 ± 9.79		
	Businessmen	20 (5.5%)	80.58 ± 9.57	3.094	0.006
	Medical Personnel	3 (0.8%)	74.96 ± 12.43		
	Teachers	23 (6.3%)	75.90 ± 13.19		
	(sth. or sb) else	150 (41.3%)	74.60 ± 10.08		
Education level	Primary and lower	146 (40.2%)	75.35 ± 10.05	0.989	0.414
	Junior high school	108 (29.8%)	75.29 ± 9.82		
	High or technical secondary school	64 (17.6%)	75.46 ± 9.82		
	Junior college or university	21 (5.8%)	75.82 ± 12.49		
	Undergraduate	24 (6.6%)	79.60 ± 10.24		
current address	City	184 (50.7%)	76.06 ± 10.23	0.396	0.673
	Suburbia	33 (9.1%)	75.96 ± 10.46		
	Countryside	146 (40.2%)	75.08 ± 9.91		
Medical examination	Semiannually	26 (7.2%)	73.33 ± 9.81	0.590	0.670
	Once a year	165 (45.5%)	75.46 ± 9.94		
	Once every two years	17 (4.7%)	77.71 ± 9.90		
	Irregularly	103 (28.4%)	76.07 ± 10.12		
	No medical check-up	52 (14.3%)	75.96 ± 10.95		

**Table 2:** The prevalence of chronic diseases in each organ system.

Disease contribution	Yes, *N* (%)
1. Heart	98 (27.20)
2. Blood vessels	156 (43.10)
3. Hematopoietic system (bone marrow)	4 (11.90)
4. Respiratory	90 (24.80)
5. Eye, ear, nose, and throat	36 (9.90)
6. Upper gastrointestinal tract	53 (14.70)
7. Lower gastrointestinal tract	67 (18.50)
8. Liver	114 (31.40)
9. Kidney	58 (16.00)
10. Urogenital	52 (14.30)
11. Motor system	102 (28.10)
12. Nervous system	96 (26.40)
13. Endocrine/Metabolic and Breast Cancer 14. Psychosis	107 (29.50) 0 (0)

### Bivariate correlation analyses

There was a moderate negative correlation between stigma and quality of life (*r* = -0.378, *P* < 0.01). There was a moderate negative correlation between depression and quality of life (*r* = -0.497, *P* < 0.01). There was a moderately positive correlation between psychological resilience and quality of life (*r* = 0.382, *P* < 0.01). There was a moderate negative correlation between psychological resilience and depression (*r* = -0.348, *P* < 0.01). There was a weak negative correlation between psychological resilience and stigma (*r* = -0.166, *P* < 0.01). There was a strong positive correlation between stigma and depression (*r* = 0.607, *P* < 0.01) (40).

Quality of life score: 75.66 ± 10.11; stigma score: 43.93 ± 15.61; depression score: 6.93 ± 4.42 (Light); and psychological resilience score: 50.22 ± 13.89. The variables’ mean, standard deviation, and correlation are displayed in **Table 3**

**Table 3:** Descriptive statistics and correlations among variables.

Variable	Mean	SD	1	2	3	4
1Stigma	43.93	15.61	1			
2Depression	6.93	4.42	0.607^**^	1		
3QOL	75.66	10.11	-0.378^**^	-0.497^**^	1	
4PR	50.22	13.89	-0.166^**^	-0.348^**^	0.382^**^	1

QOL, Quality of life; PR, Psychological resilience; SD, standard Deviation. **P < 0.01.

### The mediation analyses

We examined the mediating role of depression between stigma and quality of life, controlling for demographic information. The results showed that stigma and quality of life had a negative connection (*β* = -0.0781, *P* < 0.05), according to the data, with stigma explaining 25.89% of the quality of life (*F* = 41.80, *ΔR* = 0.2589, *P* < 0.05). There was a positive correlation between morbidity shame and depression (*β* = 0.1702, *P* < 0.001). Depression and quality of life were negatively correlated (*β* = -0.9537, *P* < 0.001). We examined the direct [*β* = -0.0781, *SE* = 0.0370, 95%CI = (-0.1509, -0.0053)] and indirect [*β* = -0.1624, *SE* = 0.0279, 95%CI = (-0.2210, -0.1133)] impacts of stigma on quality of life using 5000 repeated samplings using the Bootstrap technique. Stigma and quality of life were affected, to some extent, by depression. The direct and indirect effects of stigma on quality of life accounted for 32.45 percent and 67.55 percent of the total effect, respectively. The results are shown in **Table 4**.

**Table 4:** The mediating role of morbidity stigma on quality of life.

Variable	Depression				QOL			
	*β*	*SE*	*t*	95%CI	*β*	*SE*	*t*	95%CI
Careers	0.1557	0.0790	1.9716^*^	0.0004,0.3111	-0.2270	0.1979	-1.1469	-0.6162, 0.1622
Stigma	0.1702	0.0118	14.3932^***^	0.1470,0.1935	-0.0781	0.0370	-2.1106^*^	-0.1509, -0.0053
Depression					-0.9537	0.1314	-7.2609^***^	-1.2120, -0.6954
*R^2^*	0.3750				0.2589			
*F*	108.0055				41.80000			

*P < 0.05, ^***^P < 0.001, QOL, Quality Of Life.

### The moderation analyses

**Figure 2:**
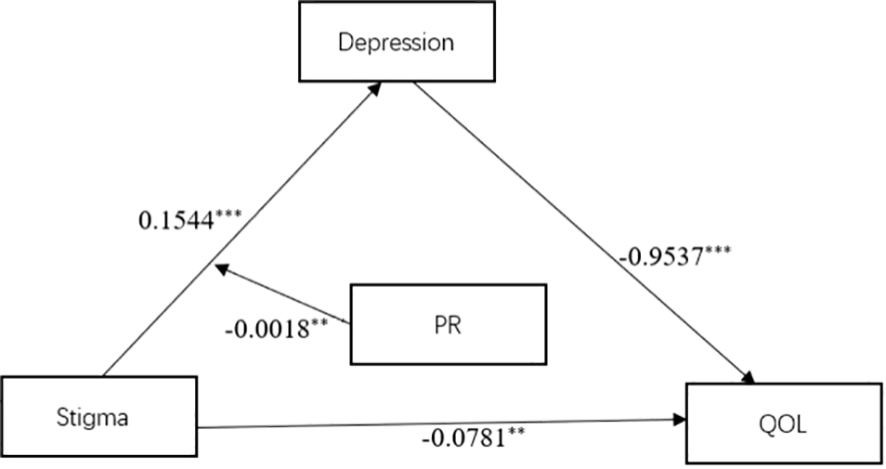
Confirmation of a model of the relationship between stigma, psychological resilience, depression and quality of life. PR, psychological resilience; QOL, Quality of life. **P < 0.01, ***P < 0.001.

We examined the moderating role of psychological elasticity using Model 7 in the moderation analysis and also calculated the parameters of the relevant model. The results of the study showed that psychological resilience [*β* = -0.0018, *SE* = 0.0008, 95%CI (-0.0034, -0.0003)] attenuated the main influence of stigma on depression (*β* = 0.1544, *SE* = 0.0115, 95%*CI* (0.1318, 0.1770). Also, the correlation model indicated the primary function of stigma in depression. The relevant results are shown in **Table 5**. **Figure 2** shows the moderated mediation model results.

**Table 5:** Mediating effects of depression between stigma and quality of life.

Variable	Effect	Boot SE	Boot LLCI	Boot ULCI	effect size (%)
Indirect effect	-0.1624	0.0279	-0.2210	-0.1133	67.55
Direct effect	-0.0781	0.0370	-0.1509	-0.0053	32.45
Total effect	-0.2404	0.0315	-0.3024	-0.1785	100.00

**Figure 3:**
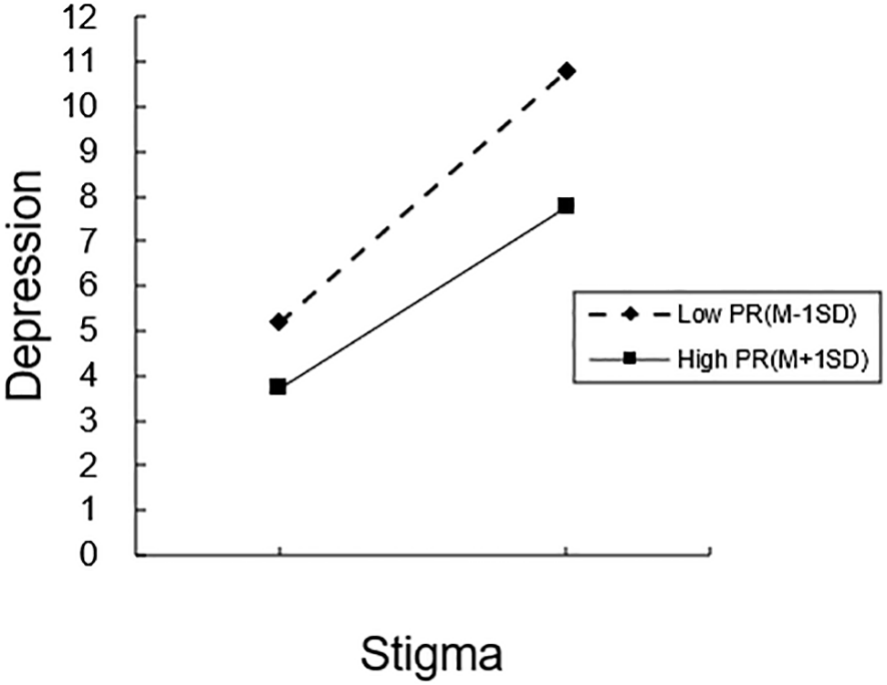
The moderating role of psychological resilience in the relationship between stigma and depression. PR, Psychological Resilience; M, Mean; SD, Standard Deviation.

An illustration of how psychological resilience mitigates the impact of stigma on depression is provided in **Figure 3**. Concerning middle-aged and senior chronic illness hospitalized patients with modest levels of PR, depression tended to rise with greater stigma, according to a straightforward slope test (*β* = 0.1796, *P* < 0.001), The overall depression score increased by 0.1796 points for every standard deviation rise in stigma; the more severe the stigma, the higher the depression. The degree of depression significantly decreased with rising stigma among middle-aged and older chronically sick inpatient patients with high PR levels (*β* = 0.1292, *P* < 0.001). The results are shown in **Table 6**.

**Table 6:** Results of the moderated mediation model analysis.

Variable	Depression				QOL			
	*β*	*SE*	*t*	95%CI	*β*	*SE*	*t*	95%CI
Careers	0.1312	0.0748	1.7543	-0.0159, 0.2783	-0.2270	0.1979	-1.1469	-0.6162, 0.1622
Stigma	0.1544	0.0115	13.4538^***^	0.1318, 0.1770	-0.0781	0.0370	-2.1106^*^	-0.1509, -0.0053
Depression					-0.9537	0.1314	-7.2609^***^	-1.2120, -0.6954
PR	-0.0803	0.0127	-6.3151^***^	-0.1054, -0.0553				
Stigma ×PR	-0.0018	0.0008	-2.3045^**^	-0.0034, -0.0003				
*R^2^*	0.4447				0.2589			
*F*	71.6673				41.8000			

^*^P<0.05, ^**^P<0.01, ^***^P<0.001, QOL, Quality of life; PR, psychological resilience.

## Discussion

This study of stigma, psychological resilience, depression, and quality of life in hospitalized middle-aged and elderly patients with chronic diseases highlights the value of increasing psychological resilience, the clinical importance of implementing effective intervention programs to improve the quality of life of patients with chronic diseases, and provides a theoretical basis for clinicians and caregivers.

According to the results, patients who experienced higher levels of stigma also had lower levels of health-related quality of life, which is in line with other studies (3, 7). Our study also suggests that depression mediates the relationship between illness stigma and quality of life. When chronically ill patients experience more negative life events and negative emotions related to chronic diseases while they are hospitalized for treatment, they develop mild depressive symptoms. If they can mobilize their internal resources and actively seek external help on time, chronically ill patients can promptly transform these stresses into social resilience and reduce the damage caused by the stresses on themselves, thus enabling the individual to maintain a positive mental health status and quality of life (41, 42). This suggests that patients with chronic diseases can reduce the level of their sense of shame through positive coping, minimizing the damage that shame can do to an individual’s physiological and psychological well-being and thus reducing the likelihood of depression occurring. According to the relevant content of behavioral therapy and anti-stigma therapy, clinical workers should carry out disease-related knowledge propaganda for patients with chronic diseases, eliminate the patients’ bad emotions, and change the patients’ bad cognition. This improves patients’ self-esteem and awareness of their illness and social value (43, 44).

Our study found that psychological resilience moderated the relationship between morbidity, shame, and depression. In particular, the relationship between illness stigma and depression was more pronounced among middle-aged and elderly chronic diseases in patients with high levels of psychological resilience. Psychological resilience can help individuals recover from adversity and adapt positively to external changes (45–47). It can also help patients with chronic diseases face the difficulties they are currently experiencing positively during their hospitalization and deal rationally with the various problems caused by their illnesses, thus reducing their level of stigma (48). In addition, enhancing the psychological resilience of patients can help them to effectively cope with various treatments and can also help them to establish good interpersonal relationships and buffer the damage caused by negative events and negative emotions due to the disease, thus reducing the level of stigma in patients with chronic diseases and decreasing the occurrence of depression and other negative emotions (49). For the elderly, who are at yet another critical stage of their lives, we need to pay attention not only to their psychological changes due to illness but also to their normal psychological needs so that they can cope successfully with physical and psychological stress. Therefore, to reduce the incidence of depression in middle-aged and elderly chronic disease inpatients, healthcare professionals should pay attention to the level of their sense of shame, strengthen mental health education, pay attention to the self-management of chronic diseases and the use of medication, help chronic disease patients correctly understand their illnesses, and actively guide chronic disease patients to return to society so that they can maintain a higher level of quality of life and a healthy psychological state.

### Limitation

The main findings of this study found that depressive symptoms mediated the relationship between stigma and quality of life, while psychological resilience moderated the relationship between stigma and depressive symptoms. These studies imply that patients with chronic illnesses who are middle-aged or older should receive a thorough assessment, that these patients report high levels of morbidity stigma, and that to lessen patients’ psychological burden and depressive symptoms, healthcare professionals should promptly offer psychological counseling to these patients (50). Meanwhile, based on cognitive-behavioral therapy (51) and well-being therapy (50, 52), appropriate psychotherapeutic approaches are adopted for chronically ill patients with migraine or systemic sclerosis to alleviate depressive symptoms and improve the patient’s adaptive capacity and quality of life. In practical terms, this study contributes to a deeper understanding of the mechanisms influencing the relationship between stigma, depressive symptoms, psychological resilience, and quality of life and provides new perspectives on improving the quality of life of patients with chronic illnesses, which should be of interest to the health sector and healthcare professionals. In addition, chronic disease patients are physiologically and psychologically more vulnerable groups; their overall quality of life level and mental health status should receive more attention, which also provides a new theoretical basis for clinical workers.

### Strength and limitations

This study of stigma, psychological resilience, depression, and quality of life in hospitalized middle-aged and elderly patients with chronic diseases highlights the value of increasing psychological resilience, the clinical importance of implementing effective intervention programs to improve the quality of life of patients with chronic diseases, and provides a theoretical basis for clinicians and caregivers.

Due to the cross-sectional design of this study, we are unable to make any causal inferences about the observed variables. At the same time, clinical features such as anxiety, mental pain, and psychosomatic syndrome may also have an impact on the quality of life of patients with chronic illnesses but have not been systematically evaluated (53–55). Therefore, future longitudinal studies should address these questions and delve into the underlying mechanisms. Secondly, only 363 cases were selected for our study, which is not representative of all chronic disease patients. Future studies may consider expanding the sample to cover more districts to increase the applicability and breadth of the service and increase the field of study by looking at specific setting factors in a multilevel model (56). Healthcare workers should provide specialized services not only to inpatients but also to outpatients and patients treated in rehabilitation clinics. Improving the assessment of middle-aged and elderly patients with chronic diseases and improving planned interventions improves the quality of life of middle-aged and elderly patients with chronic diseases.

## Conclusions

Future research should employ additional longitudinal studies to characterize the route links between the variables, as the current cross-sectional study was unable to establish a causal relationship between the variables. Meanwhile, only 363 subjects were selected for this study due to time, human, and material resources, which cannot represent the quality of life level of chronic disease patients nationwide. Future studies should expand the sample size for research. This study, as a cross-sectional study, limits the ability to test for causality and has not yet assessed the impact of some clinical features such as anxiety, physical and psychological syndromes, etc., which can also have an impact on quality of life, which are some of the research gaps that currently exist in this study. Future studies should incorporate a systematic assessment of more factors that affect the quality of life of patients with chronic diseases.

## Data availability statement

The original contributions presented in the study are included in the article/Supplementary Material. Further inquiries can be directed to the corresponding author.

## Ethics statement

Approval for this study was given by the medical ethics committee of Wannan Medical College (approval number 2021– 3) and written informed consent was obtained from the participants. All methods were performed following the Declarations of Helsinki.

## Author contributions

QJ: Data curation, Writing – original draft, Writing – review & editing. LZ: Data curation, Writing – original draft, Writing – review & editing. JX: Data curation, Writing – review & editing. PJ: Data curation, Writing – review & editing. MS: Data curation, Writing – review & editing. YC: Data curation, Writing – review & editing. LG: Data curation, Writing – review & editing.
